# Integrative Analysis of Pyroptosis-Related Prognostic Signature and Immunological Infiltration in Lung Squamous Cell Carcinoma

**DOI:** 10.1155/2022/4944758

**Published:** 2022-06-01

**Authors:** Pengfei Luo, Yongjun Jiang, Sijuan Ding, Shaohui Jiang, Ruoting Tang, Zhaohui Tang

**Affiliations:** Department of Oncology, The Central Hospital of Yongzhou, No. 396 Yiyun Road, Yongzhou City, 425000 Hunan Province, China

## Abstract

**Background:**

Lung cancer is one of leading causes of human health threatening with approximately 2.09 million initially diagnosed cases and 1.76 million deaths worldwide annually. Pyroptosis is a programmed cell death mediated by Gasdermin family proteins. Pyroptosis could suppress the tumor oncogenesis and progression; nevertheless, pyroptosis could promote tumor growth by forming a suitable microenvironment.

**Methods:**

LASSO Cox regression analysis was performed to construct prognostic pyroptosis-related gene (PRG) signature. A ceRNA was constructed to explore the potential lncRNA-miRNA-mRNA regulatory axis in LUSC.

**Results:**

The expression of 26 PRGs were increased or decreased in LUSC. We also summarized simple nucleotide variation and copy number variation landscape of PRGs in LUSC. Prognosis analysis suggested a poor overall survival rate in LUSC patients with high expression of IL6, IL1B, ELANE, and CASP6. A pyroptosis-related prognostic signature was developed based on four prognostic PRGs. High-risk score LUSC patients had a poor overall survival rate versus low-risk score patients with an AUC of 0.565, 0.641, and 0.619 in 1-year, 3-year, and 5-year ROC curves, respectively. Moreover, the risk score was correlated with immune infiltration in LUSC. Further analysis revealed that pyroptosis-related prognostic signature was correlated with immune cell infiltration, tumor mutation burden, microsatellite instability, and drug sensitivity. We also constructed a ceRNA network and identified a lncRNA KCNQ1OT1/miR-328-3p/IL1B regulatory axis for LUSC.

**Conclusion:**

A bioinformatics method was performed to develop a pyroptosis-related prognostic signature containing four genes (IL6, IL1B, ELANE, and CASP4) in LUSC. We also constructed a ceRNA network and identified a lncRNA KCNQ1OT1/miR-328-3p/IL1B regulatory axis for LUSC. Further in vivo and in vitro studies should be conducted to verify these results.

## 1. Introduction

Lung cancer is one of leading causes of human health threatening with approximately 2.09 million initially diagnosed cases and 1.76 million deaths worldwide annually [1]. Lung squamous cell carcinoma (LUSC) is one of most frequent pathological subtypes of lung cancer [2]. LUSC was characterized by large blood vessels and proximal bronchus invasion, leading to hemoptysis. Without the opportunity of radical surgery, over 50% the initially diagnosed LUSC patient would lose their lives within 12 months [3]. Moreover, molecular mechanism for LUSC had not been elucidated and the therapies were limited, resulting in a poor prognosis of LUSC and the average 5-year overall survival (OS) rate of 10-20% [4]. These data demonstrated the urgent need to clarify potential mechanism of LUSC, thus identifying the prognostic biomarkers and therapy targets for LUSC.

Pyroptosis is a programmed cell death mediated by Gasdermin family proteins [5]. Different from autophagy and apoptosis, pyroptosis was characterized by unique morphology and mechanism [6]. Releasing inflammatory cytokines IL-1*β* and IL-18, pyroptosis played a crucial function in the pathogenesis of many diseases, including atherosclerosis, sepsis, and Parkinson's disease [7–9]. Recent studies found that pyroptosis suppressed the tumor oncogenesis and progression; nevertheless, pyroptosis could promote tumor growth by forming a suitable microenvironment [10]. Increasing evidence suggested pyroptosis as a new frontier for tumor due to its effect on the proliferation, invasion, and metastasis of tumor [11]. Moreover, the pyroptosis-related signature could serve as a prognosis biomarker and predicts immune microenvironment infiltration in certain type of cancer, including ovarian cancer, gastric cancer, and lung adenocarcinoma [12–14].

Given the existing evidences, we suspect that pyroptosis may also be involved in the oncogenesis and progression of LUSC. However, the prognostic value and potential mechanism of pyroptosis in LUSC have not be fully studied. The current study constructed a pyroptosis-related prognosis signature using the LASSO-Cox analysis. Moreover, we also explored the correlation between pyroptosis-related prognosis signature and the immune microenvironment in LUSC. A ceRNA network was constructed to clarify potential regulatory axis in LUSC.

## 2. Materials and Methods

### 2.1. Dataset Acquisition and Preprocessing

Gene expression (FPKM) and simple nucleotide variation of LUSC patients and clinic information data were downloaded from the Cancer Genome Atlas (TCGA, https://portal.gdc.cancer.gov/) on October 3, 2021. FPKM data were then normalized to transcripts per kilobase million value. Using UCSC Xena (https://xena.ucsc.edu/), we isolated copy number variation (CNV) data. R (version 4.0.5) with R packages was applied to perform dataset processing.

### 2.2. Defining the Expression and Genetic Alteration Landscape of Pyroptosis-Related Genes (PRGs)

Based on a previous literature, we obtained 33 PRGs (Supplementary Table 1) [13, 15]. The “limma” package was applied for identification of differently expressed pyroptosis-related genes (PRGs) and a *p* value was set as 0.05. The mutation landscape of PRGs was visualized using waterfall function within the “maftools” package. By using “RCircos” package in R, we presented the location of CNV alteration of PRG human chromosomes.

### 2.3. Gene Ontology (GO) and Kyoto Encyclopedia of Genes and Genomes (KEGG) Pathway Analysis

Using differently expressed pyroptosis-related genes (PRGs) in LUCS, we then performed GO and KEGG pathway analysis with “ggplot2” package in R with a *p* < 0.05. Noteworthily, GO analysis comprises biological process (BP), cellular component (CC), and molecular function (MF).

### 2.4. Construction of a Pyroptosis-Related Prognostic Signature

A Kaplan-Meier method was used to identify the prognostic PRGs with *p* values and hazard ratio (HR) with 95% confidence interval (CI) calculated by log-rank test. The LASSO Cox regression model was applied to isolate the candidate genes and construct a pyroptosis-related prognostic signature based on these prognostic PRGs. The risk score of each LUSC patients was calculated using the formula as follows: risk score = ∑_*i*_^4^*Xi* × *Yi* (*X*: coefficients, *Y*: candidate gene expression). With the median risk score as the cutoff, all LUSC cases were divided into low- and high-risk subgroups. The difference of OS rate in two subgroups of LUSC was calculated with Kaplan-Meier survival curves, and time-dependent receiver operating characteristic curves (ROC curves) were applied to evaluate the efficiency of the prognostic signature with “timeROC” packages. Considering the clinical characters and prognostic signature, we performed univariate and multivariate cox regression and the result was drawn in forest with “forestplot” R package. Based on the result, we constructed a predicted nomogram to predict the 1-, 3-, and 5-year OS rate of LUSC patients.

### 2.5. Immune Infiltration, Drug Sensitivity, TMB, and MSI Analysis

The abundance of immune cells was isolated from TIMER database (https://cistrome.shinyapps.io/timer/). We collected the IC50 of 265 small molecules in 860 cell lines, and its corresponding mRNA gene expression from the Cancer Therapeutics Response Portal (CTRP). The mRNA expression data and drug sensitivity data were merged. The microsatellite instability (MSI) score of LUSC patients was calculated as described previously [16]. After obtaining the masked somatic mutation file (varscan. Somatic. Maf) of LUSC patients from TCGA, we calculated the TMB score of LUSC patients with the “maftools” package in R. Pearson's correlation analysis was used to performed to calculate the correlation between pyroptosis-related prognostic signature and immune infiltration, drug IC50, TMB, and MSI score. A *p* value < 0.05 was considered statistically significant difference.

### 2.6. Construction of Potential Regulatory Axis

We then constructed a PPI network with String (https://string-db.org/) to identify hub gene among pyroptosis-related prognostic signature. This was followed by identification of the miRNA target of hub gene using miRDB (http://mirdb.org/), StarBase (http://starbase.sysu.edu.cn/), and miRWalk (http://mirwalk.umm.uni-heidelberg.de/). After identifying miRNA targets of hub genes, we used StarBase (http://starbase.sysu.edu.cn/) and LncBase module of DIANA tool (http://carolina.imis.athena-innovation.gr/) to predict lncRNA target interacting with miRNA. Moreover, the expression of miRNA and lncRNA was detected with Student's *t*-test using TCGA LUSC dataset. A *p* value < 0.05 was considered statistically significant difference.

## 3. Results

### 3.1. Expression and Genetic Mutation Landscape of PRGs in LUSC

**Figure 1:**
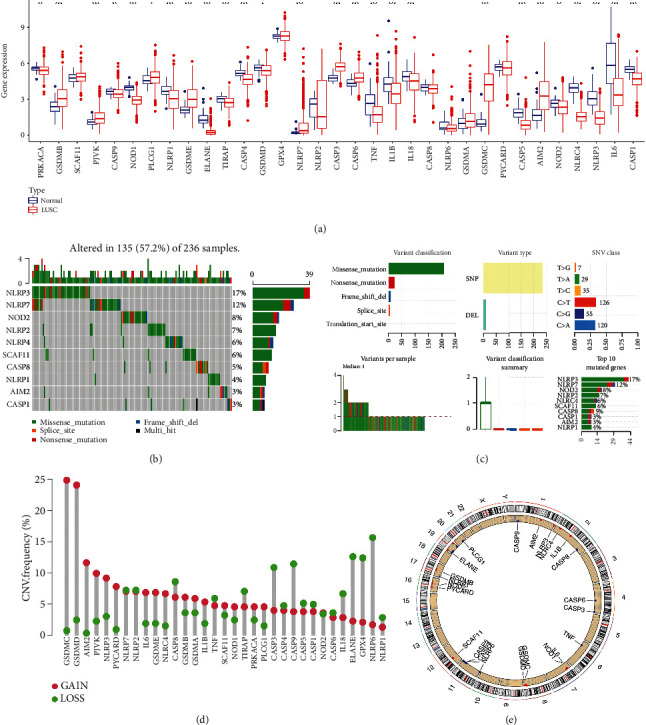
Gene expression and genetic mutation landscape. (a) The mRNA level of PRGs in LUSC versus lung tissues. (b, c) The simple nucleotide variation of PRGs in LUSC. (d, e) The CNV variation frequency and chromosomes location of PRGs in LUSC. ^∗^*p* < 0.05;  ^∗∗^*p* < 0.01;  ^∗∗∗^*p* < 0.001; PRG: pyroptosis-related gene; LUSC: lung squamous cell carcinoma; CNV: copy number variation.

Figure 1(a) shows the expression landscape of PRGs in LUSC and the expression of 26 PRGs were increased or decreased in LUSC (*p* < 0.05). Minutely, upregulation was obtained in the expression of GSDMB, PJVK, PLCG1, GSDME, NLRP7, CASP3, CASP6, GSDMC, and AIM2 while downregulation was obtained in the expression of PRKACA, CASP9, NOD1, NLRP1, ELANE, TIRAP, CASP4, GSDMD, TNF, IL1B, IL18, CASP5, NOD2, NLRC4, NLRP3, IL6, and CASP1 in LUSC (all *p* < 0.05). Simple nucleotide variation of PRGs in LUSC cases is shown in Figures 1(b) and 1(c), revealing that 135 of 236 (57.2%) LUSC samples presented with simple nucleotide variation and NLRP3 was the gene with the highest frequency of mutation followed by NLRP7 and NOD2. We found that missense mutation ranked the top variant classification and C>A was the most common SNV class (Figure 1(c)). In CNV analysis, the data suggested that more than half of 33 PRGs had copy number amplification while the other had a widespread CNV deletion (Figure 1(d)). The location of CNV alteration of PRGs on human chromosomes is shown in Figure 1(e).

### 3.2. GO and KEGG Pathway Analysis

**Figure 2:**
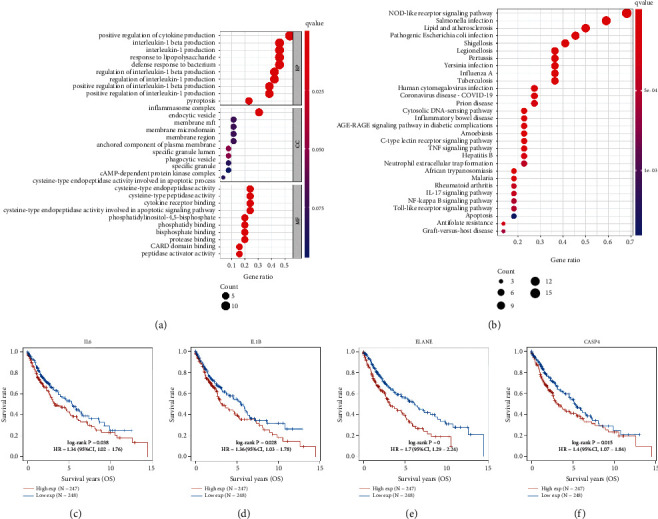
The functional enrichment and prognostic analysis. (a, b) The enriched items in gene ontology and Kyoto Encyclopedia of Genes and Genomes analysis. (c–f) Overall survival curve in LUSC patients with high/low expression of IL6, IL1B, ELANE, and CASP4. LUSC: lung squamous cell carcinoma; BP: biological process; CC: cellular component; MF: molecular function.

GO and KEGG pathways were performed with above 26 differently expressed PRGs. The result of GO analysis in Figure 2(a) indicated that these PRGs were enriched in positive regulation of cytokine production, pyroptosis, endocytic vesicle, cysteine-type endopeptidase activity, cytokine receptor binding, and CRAD binding. As for KEGG pathways analysis, the data in Figure 2(b) suggested that these PRGs were enriched in NOD-like receptor signaling pathway, cytosolic DNA-sensing pathway, NF-KB signaling pathway, and apoptosis.

### 3.3. Construction of a Pyroptosis-Related Prognostic Signature

**Figure 3:**
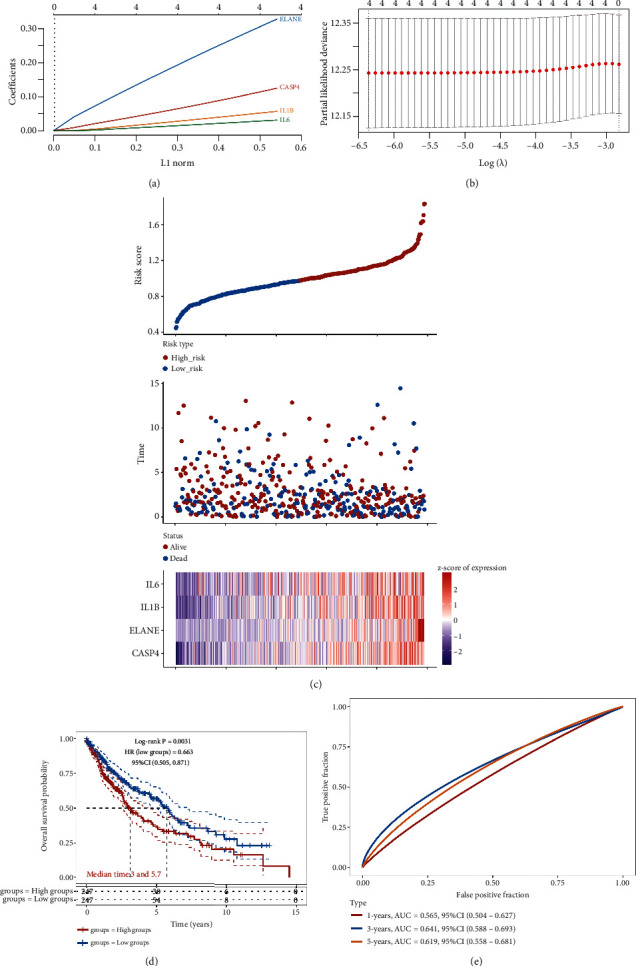
Construction of a pyroptosis-related prognostic signature. (a, b) The coefficient and partial likelihood deviance of prognostic signature. (c) The risk score distribution, survival status of LUSC cases and gene expression profile of this prognostic signature. (d, e) Overall survival curve in the high-/low-risk group and the ROC curve evaluating prognosis predicting performance of LUSC patients.

**Figure 4:**
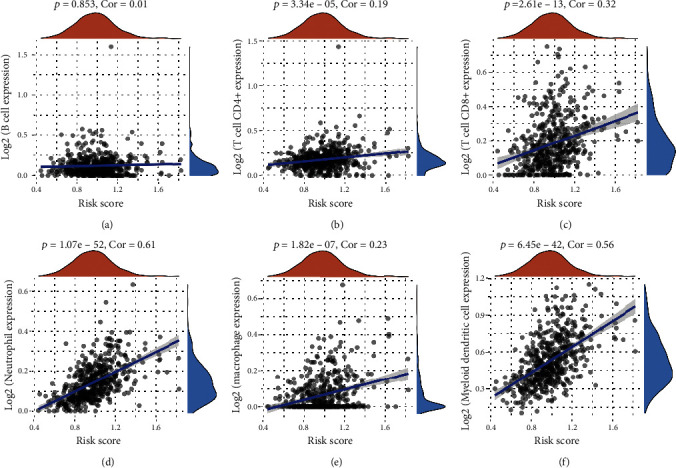
Risk score correlated with immune infiltration in LUSC. The correlation between risk score and the expression of B cells (a), CD4+ T cells (b), CD8+ T cells (c), neutrophils (d), macrophage (e), and dendritic cells (f).

Kaplan-Meier survival curves revealed that LUSC patients with high expression of IL6 (*p* = 0.038, HR = 1.34), IL1B (*p* = 0.028, HR = 1.36), ELANE (*p* < 0.001, HR = 1.7), and CASP4 (*p* = 0.015, HR = 1.4) had a poor OS rate versus low expression group (Figures 2(c)–2(f)). LASSO cox regression analysis was conducted to develop a pyroptosis-related prognostic signature using above four genes. The coefficient and partial likelihood deviance of prognostic signature are shown in Figures 3(a) and 3(b).  Figure 3(c) shows the risk score distribution, survival status of LUSC cases and gene expression profile of this prognostic signature. The risk score of LUSC patients was calculated with a formula: risk score = (0.0312)∗IL6 + (0.0569)∗IL1B + (0.3276)∗ELANE + (0.1244)∗CASP4. All LUSC cases were divided into the high- and low-risk groups. As expected, high risk score patients had a poor OS rate versus low risk score patients with a median time of 3 vs. 5.7 years (Figure 3(d), p =0.0031). As shown in Figure 3(e), the AUC was 0.565, 0.641, and 0.619 in 1-year, 3-year, and 5-year ROC curves, demonstrating that this prognostic signature had a good performance in predicting the prognosis of LUSC patients. Moreover, further analysis revealed that immune infiltration level of CD4+ T cells (*p* = 3.34*e* − 5, Figure 4(b)), CD8+ T cells (*p* = 2.61*e* − 13, Figure 4(c)), neutrophils, (*p* = 1.07*e* − 52, Figure 4(d)), macrophage (*p* = 1.82*e* − 7, Figure 4(e)), and dendritic cells (*p* = 6.45*e* − 42, Figure 4(f)) was positively correlated with the risk score of LUSC patients.

### 3.4. Construction of a Predictive Nomogram

**Figure 5:**
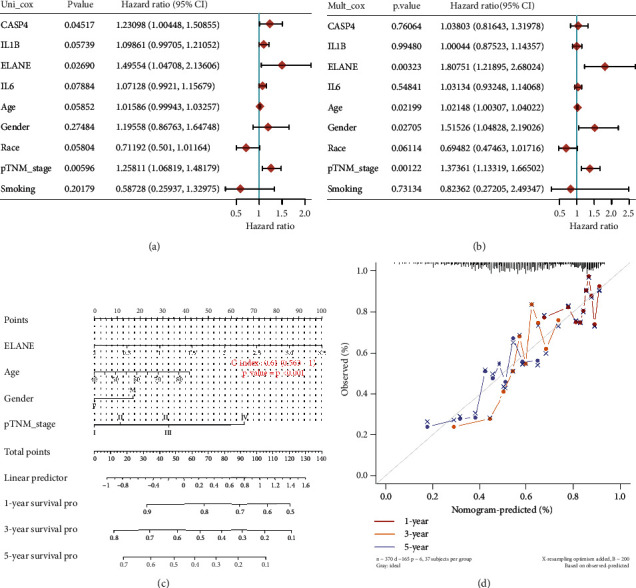
Construction of predictive nomogram. (a, b) Univariate and multivariate cox regression considering clinical parameters and pyroptosis-related prognostic signature. (c, d) Predictive nomogram to predict the 1-year, 3-year, and 5-year overall survivals of LUSC patients. Calibration curve for the overall survival nomogram model in the discovery group. A dashed diagonal line represents the ideal nomogram.

Considering clinicopathologic features and prognostic signature, univariate and multivariate analyses were performed to identify the prognostic factors. As shown in Figures 5(a) and 5(b), the result indicated ELANE, age gender, and pTNM stage are independent prognosis factors for LUSC patients. Based on these data, we then constructed a predictive nomogram, which indicated that this predictive nomogram could predict relatively well in the 3-year and 5-year OS rates compared with an ideal model in the entire cohort (Figures 5(c) and 5(d)).

### 3.5. PRGs Correlated with Immune Infiltration in LUSC

**Figure 6:**
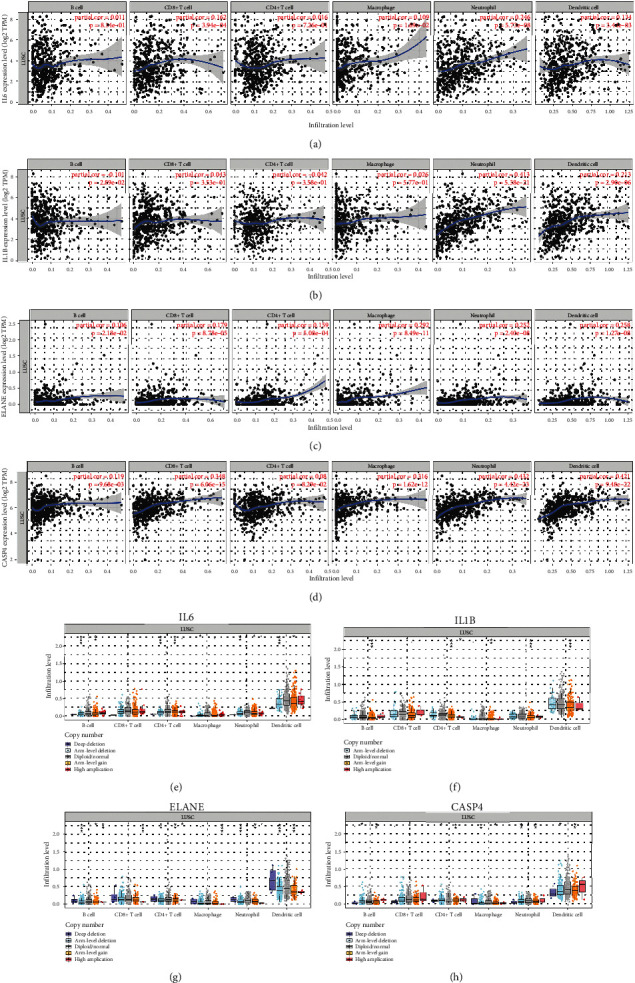
Pyroptosis-related prognostic signature correlated with immune infiltration in LUSC. The correlation between immune cells expression and the mRNA level of IL6 (a), IL1B (b), ELANE (c), and CASP4 (d) in LUSC. The correlation between different copy number alteration of IL6 (e), IL1B (f), ELANE (g), and CASP4 (h) and immune cells expression and in LUSC. ^∗^*p* < 0.05;  ^∗∗^*p* < 0.01;  ^∗∗∗^*p* < 0.001.

The above result revealed a significant correlation between risk score and immune infiltration. We further explored with correlation between four PRGs and immune infiltration in LUSC. As expected, there was a positive correlation between IL6 and the immune abundance of CD8+ T cell (cor = 0.162), macrophage (cor = 0.109), neutrophils (cor = 0.246), and dendritic cell (cor = 0.135) (Figure 6(a), *p* < 0.05). IL1B expression showed negative correlation with B cells (cor = −0.101) and positive correlation with neutrophils (cor = 0.413) and dendritic cell (cor = 0.213) (Figure 6(b), *p* < 0.05). The abundance of B cells, CD8+ T cells, CD4+ T cells, macrophage, neutrophils, and dendritic cells increased as the expression of ELANE and CASP4 increased (Figures 6(c) and 6(d)). Moreover, we also found that certain SCNA of these PRGs could inhibit immune infiltration in LUSC (Figures 6(e)–6(h)).

### 3.6. The Correlation between PRG Expression and TMB, MSI, and Drug Sensitivity

**Figure 7:**
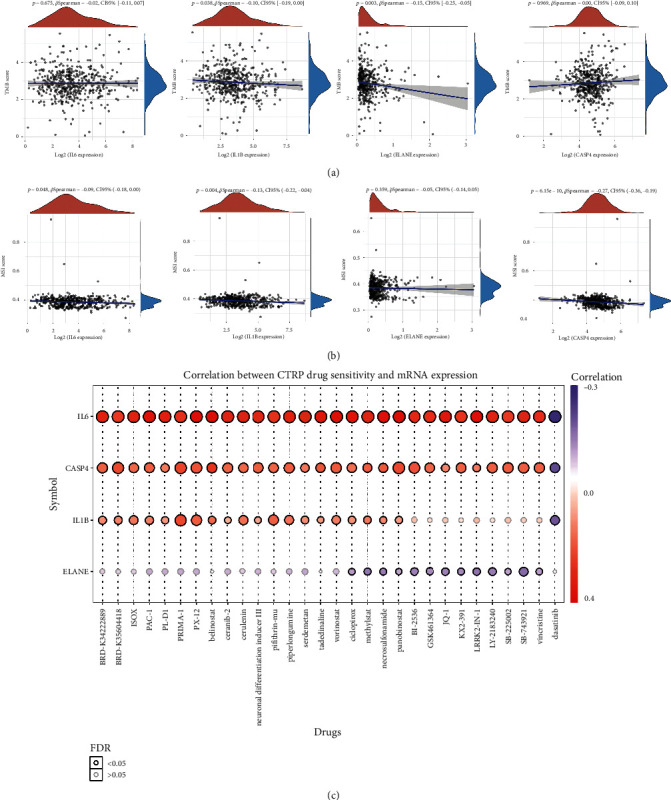
Pyroptosis-related prognostic signature correlated with TMB, MSI, and drug sensitivity in LUSC. (a) The correlation between pyroptosis-related prognostic signature and TMB. (b) The correlation between pyroptosis-related prognostic signature and MSI. (c) The correlation between pyroptosis-related prognostic signature and drug IC50 of CTRP. TMB: tumor mutation burden; MSI: microsatellite instability; CTRP: cancer therapeutics response portal.

**Figure 8:**
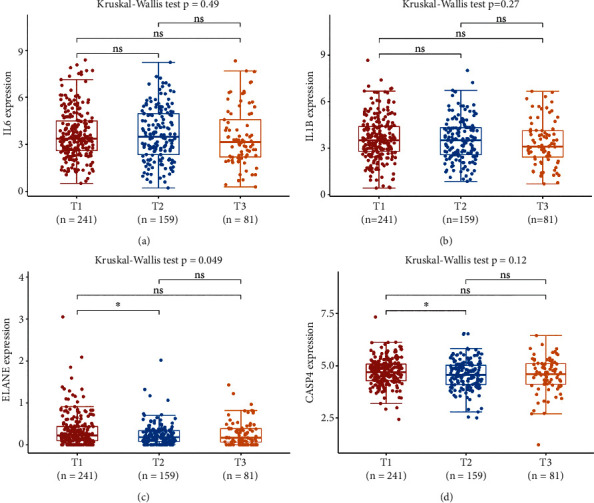
The expression of pyroptosis-related prognostic signature in different TNM stage in LUSC patients. The expression of IL6 (a), IL1B (b), ELANE (c), and CASP4 (d) in different TNM stages in LUSC patients. ^∗^*p* < 0.05.

Increasing evidences suggested TMB as a predictive marker for immunotherapy efficacy in lung cancer [17, 18]. MSI was also referred as a predictive marker for cancer immunotherapy [19]. To clarify the important role of PRGs in LUSC, we then explored its correlation with TMB and MSI in LUSC. As shown in Figure 7(a), the TMB score decreased as the expression of IL1B (*p* = 0.038) and ELANE (*p* = 0.003) increased. Similarly, the MSI score decreased as the expression of IL6 (*p* = 0.048), IL1B (*p* = 0.004), and CASP4 (*p* = 6.15*e* − 10) increased (Figure 7(b)). To develop a therapy target, one of vital way is to analyze its correlation with exited drugs. Interestingly, drug sensitivity analysis indicated high expression of IL6, CASP4, and IL1B was correlated with drug resistance of CTRP (Figure 7(c)), suggesting that IL6, CASP4, and IL1B may serve as the potential biomarkers for drug scanning. We also explored PRGs expression in different TNM stages of LUSC patients. However, only ELANE showed positive correlation with TNM stage (Figure 8(c), *p* = 0.049). There is no significant difference between the expression of IL6 (Figure 8(a)), IL1B (Figure 8(b)), and ELANE (Figure 8(d)) and different TNM stage LUSC patients (all *p* > 0.05).

### 3.7. Construction of a lncRNA-miRNA-mRNA Regulatory Axis

**Figure 9:**
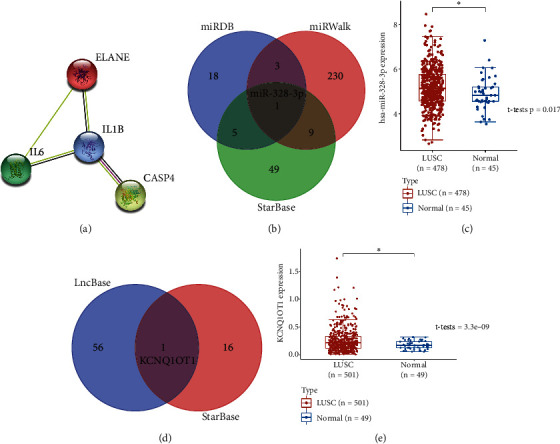
Construction of lncRNA-miRNA-mRNA regulatory axis. (a) PPI network identifying hug gene. (b) The miRNA targets predicted by miRDB, miRWalk, and StarBase. (c) The expression of miR-328-3p in LUSC. (d) The lncRNA targets predicted by lncBase and StarBase. (e) The expression of lncRNA KCNQ1OT1 in LUSC. ^∗^*p* < 0.05.

A PPI network was constructed and revealed IL1B as the hub gene among pyroptosis-related prognostic signature (Figure 9(a)), and we selected IL1B for further analysis. The miRNA target of IL1B was obtained from miRDB, miRWalk, and StarBase. As a result, miR-328-3p was suggested as the miRNA target for IL1B (Figure 9(b)). Moreover, we also found that miR-328-3p was upregulated in LUSC (Figure 9(c), *p* = 0.017). We further explored the lncRNA target of miR-328-3p, and the data from lncBase and StarBase suggested lncRNA KCNQ1OT1 as a lncRNA target interacting to miR-328-3p (Figure 9(d)). Further analysis demonstrated upregulation of KCNQ1OT1 in LUSC versus normal tissues (*p* = 3.3*e* − 9, Figure 9(e)). Thus, lncRNA KCNQ1OT1/miR-328-3p/IL1B regulatory axis may play a vital role in the progression in LUSC. Further in vivo and in vitro studies should be conducted to verify this hypothesis.

## 4. Discussion

Pyroptosis was Gasdermin-mediated programmed necrosis associated with pathogenesis of many diseases [20]. Pyroptosis can be chemically induced in tumor cells in the absence of any bacterial or viral infection [21]. Pyroptosis was involved in all stages of carcinogenesis, suggesting it as one of most promising directions for cancer research [22]. Moreover, the pyroptosis-related signature could serve as a prognosis biomarker and predicts immune microenvironment infiltration in a certain type of cancer, including ovarian cancer and gastric cancer [12, 13]. However, the role of pyroptosis in LUSC was not fully established.

Expression analysis revealed upregulation of GSDMB, PJVK, PLCG1, GSDME, NLRP7, CASP3, CASP6, GSDMC, and AIM2 while revealing downregulation of PRKACA, CASP9, NOD1, NLRP1, ELANE, TIRAP, CASP4, GSDMD, TNF, IL1B, IL18, CASP5, NOD2, NLRC4, NLRP3, IL6, and CASP1 in LUSC versus normal tissues. Kaplan-Meier survival curves revealed that LUSC patients with high expression of IL6, IL1B, ELANE, and CASP4 had a poor OS rate versus a low-expression group. Actually, these genes were suggested as a prognosis biomarker for lung cancer or other types of cancers. IL-6 polymorphism was associated with survival prognosis of non-small-cell lung cancer (nSCLC) [23]. A genomic analysis revealed that high ELANE expression in LUAD was associated with a good prognosis [24]. In esophageal squamous cell carcinoma, CASP4 served as a prognostic biomarker and is associated with poor prognosis [25].

Based on the above four prognostic PRGs, LASSO Cox regression analysis was conducted to develop a pyroptosis-related prognostic signature, which could serve as a prognosis biomarker in LUSC and predict the OS rate with medium to high accuracy. We also constructed a predictive nomogram, which could predict relatively well in the 3-year and 5-year OS rates compared with an ideal model in the entire cohort. Actually, a previous study revealed that pyroptosis-related signature could serve as a prognosis biomarker in a certain type of cancer. Ying et al. constructed a 7-pyroptosis-related signature, which could predict the prognosis of ovarian cancer [13]. Another pyroptosis-related signature could serve as a biomarker in gastric cancer, for predicting prognosis and immune microenvironment infiltration [12]. In our study, we firstly identified a pyroptosis-related prognostic gene signature for LUSC, further confirming the important role of pyroptosis for the development and prognosis of cancer.

Our results revealed that risk score and PRGs (IL6, IL1B, ELANE, and CASP4) were significant correlated with immune infiltration. Previous study had revealed the vital role of these PRGs in tumor microenvironment and immune infiltration. Combined blocking of IL-6 and PD-1/PD-L1 signals can eliminate their immunosuppressive effects in the tumor microenvironment [26]. In colorectal cancer, macrophage-derived IL6 in immune microenvironment was associated with chemoresistance [27]. In lung cancer, IL6 was involved in cell autonomous propensity for metastasis and establishing the metastatic niche [28]. Interestingly, our study also found that high IL6 expression was correlated with drug resistance in LUSC. Thus, the immune-microenvironment may confer chemoresistance of LUSC through immune cell derived IL6. A further study should be conducted to clarify the molecular mechanism of this regulatory axis.

Another vital finding of the current study was that we identified a lncRNA KCNQ1OT1/miR-328-3p/IL1B regulatory axis in LUSC by developing a ceRNA network. Zhang et al. found that KCNQ1OT1 was upregulation in non-SCLC and associated with clinicopathology [29]. Another study revealed that KCNQ1OT1 was a prognostic biomarker in non-SCLC and could accelerate tumor progression by the regulation of miR-204-5p/ATG3 axis [30, 31]. Moreover, miR-328-3p could accelerate the occurrence and progression of lung cancer via NF2-mediated Hippo axis [32]. High expression of miR-328-3p was correlated with radiotherapy sensitivity in non-SCLC [33]. Our study revealed another lncRNA KCNQ1OT1/miR-328-3p/IL1B regulatory axis in LUSC, which may also play a vital function in the tumor progression.

Pyroptosis was a double-edged sword and played a vital function in both tumorigenesis and antitumor immunities at all stages of tumor development [34]. Different from autophagy and apoptosis and necroptosis, pyroptosis was characterized by unique morphology and mechanism [6, 35]. Increasing evidences revealed that pyroptosis was involved in host defense and highly correlated with bridging innate and adaptive immunity [36]. Moreover, targeting pyroptosis and developing related drugs may provide another immunotherapy strategy for cancer [36]. Our study clarified a significant correlation between pyroptosis-related prognostic signature and immunological infiltration in LUSC, providing some data for the pyroptosis-related immunotherapy of LUSC.

In conclusion, a bioinformatics method was performed to develop a pyroptosis-related prognostic signature containing four genes (IL6, IL1B, ELANE, and CASP4) in LUSC. We also constructed a ceRNA network and identified a lncRNA KCNQ1OT1/miR-328-3p/IL1B regulatory axis for LUSC. Further in vivo and in vitro studies should be conducted to verify these results.

## Data Availability

The analyzed datasets generated during the study are available from the corresponding author on reasonable request.

## Supplementary Materials

Supplementary Materials Supplementary Table 1: 33 pyroptosis-related genes obtained from a previous study.
